# Supplementary material for: Control of plant cell fate transitions by transcriptional and hormonal signals
Source: eLife. 2017 Oct 23;6:e30135. doi: 10.7554/eLife.30135 (PMC5693117; doi:10.7554/eLife.30135)
Supplement: Supplementary file 3: [file elife-30135-supp3.pdf]

Control of plant cell fate transitions by transcriptional and hormonal signals

Computational Model

The aim of our computational modeling is twofold. First, we seek to understand the relative contribution of cell proliferation, fate transition and respecification in mediating the *pCLV3:HEC1-GR* phenotype. We use the model to check which hypothetical scenarios are compatible with our experimental observations. Second, we aim to get insights into the *hec1,2,3* phenotype which admits only limited experimental manipulations. Taking into account the functions of HEC derived from experiments and simulations of HEC-gain-of-function scenarios, we use the model to conclude how changes in cell proliferation, fate transition and respecification might contribute to the *hec1,2,3* mutant phenotype.

Assumptions

We develop a mathematical model describing dynamics of different cell populations in the SAM with a simplified geometry. We make the following assumptions.

1. The shoot apical meristem (SAM) and the central zone (CZ) have a circular shape. We assume that cells in all zones of the meristem have the same size.

2. The CZ consists of stem cells. The peripheral zone (PZ) consists of incipient primordia and of unspecified PZ cells. As incipient primordia, we understand regions inside the PZ consisting of cells that are committed to organ formation.

3. Cells located in the CZ divide slower than cells located in the PZ [1]. For simplicity, we assume that all PZ cells divide at the same rate.

4. Incipient primordia are initiated at the central boundary of the PZ. The frequency of primordia initiation may depend on the size of the CZ.

5. A fixed time after initiation, incipient primordia separate from the meristem and form organs. Unspecified PZ cells contribute to longitudinal growth of the plant. Unspecified cells at the peripheral boundary of the SAM leave the SAM with a constant rate.

6. We consider only the L1 layer of the SAM. We assume that cells cannot transit from L1 to other layers of the meristem or vice versa.

Model structure

The developed model consists of a system of ordinary differential equations (ODEs). The equations describe the evolution in time of different SAM cell populations. These are the stem cells in the CZ, multiple incipient primordia in the PZ and unspecified PZ cells at different locations.

Due to the circular geometry, stem cell counts and total cell counts can be linked to radius and circumference of the CZ and the SAM respectively. Using the average cell diameter, the number of cells located along the circumference of the CZ, along the central boundary of the PZ or along the peripheral boundary of the SAM can be approximated. This approach allows to model independently cells located along the boundary of meristem structures and cells located in their centers. Important parameters and variables are listed in Table 1.

The biological hypotheses we study are formulated in terms of cell proliferation, fate transition and re-specification rates, i.e., average quantities describing behavior of meristem cells. Using ordinary differential equations such quantities can be straightforwardly linked to observable meristem dynamics in terms of cell counts, which are measured in our experiments. Therefore, we choose a population based model which is formulated in the framework of ODEs. Advantages of the ODE approach lie in its simplicity, in a relatively small number of parameters and in fast numerical simulations.

Dynamics in the CZ

Denote by $n_{CZ}(t)$ the number of CZ cells at time t, by a_{CZ}, we denote the area occupied by an average CZ cell. The total area of the CZ at time t, $A_{CZ}(t)$, is given by $A_{CZ}(t) = n_{CZ}(t)a_{CZ}$. Since the CZ has a circular shape, its radius at time t, $R_{CZ}(t)$, is given by

$$R_{CZ}(t) = \sqrt{A_{CZ}(t)/\Pi} = \sqrt{n_{CZ}(t)a_{CZ}/\Pi}.$$

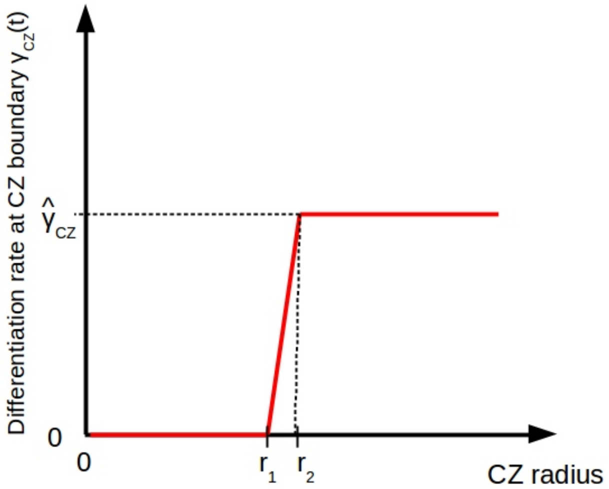

Figure 1: Transition rate γ_{CZ}. Cells at the periphery of the CZ transit to the PZ fate at the rate γ_{CZ}. The rate depends on the radius of the CZ, i.e., if the CZ cell position is near the center, its probability to become a PZ cell is low compared to a CZ cell located distant from the center. The shape of γ_{CZ} models the function of WUS. WUS induces stem cell fate and is produced in the OC located below the center of the CZ. For simplicity, γ_{CZ} is taken to be piece-wise linear. The maximal rate $\hat{\gamma}_{CZ}$ corresponds to the rate of fate transition in absence of WUS.

The population dynamics in the CZ are governed by proliferation and fate transition. We neglect death of cells residing in the CZ. Cell proliferation is quantified by the proliferation rate p_{CZ} which describes the frequency of cell divisions per unit of time. For simplicity, we assume that all cells inside the CZ divide at the same rate p_{CZ}.

In accordance with our experimental observations CZ cells can only adopt the PZ fate if they are located at the periphery of the CZ. We quantify fate transition by the transition rate γ_{CZ}. The rate γ_{CZ} describes which fraction of the peripheral CZ cells adopts PZ fate per unit of time.

Maintenance of the CZ fate depends on the presence of certain signals, such as WUS. Fate transition from the CZ to the PZ fate occurs if the level of these signals decreases [3,4]. WUS is produced by cells localized in the organizing center OC and then transported to the CZ [5]. To model the impact of the WUS distribution observed in a wild type SAM, we assume that the fate transition rate of cells located at the outer boundary of the CZ increases with increasing meristem radius, see Figure 1.

For simplicity, we assume that the fate transition rate γ_{CZ} is zero if the radius of the CZ is below a given value r_1. If the radius of the CZ is above a certain value r_2, the fate transition rate is equal to a constant $\hat{\gamma}_{CZ}$ that is larger than the proliferation rate. The value of $\hat{\gamma}_{CZ}$ corresponds to the fate transition rate in absence of WUS. Between r_1 and r_2 the fate transition rate is assumed to increase linearly, see Figure 1. This choice of γ_{CZ} follows the observation that CZ cell fate depends on the WUS concentration: if WUS concentration is above a certain threshold, the CZ cells maintain stem cell fate and if it is below a certain threshold, the CZ cells transit towards PZ fate.

We approximate the diameter of a cell by $\delta := \sqrt{a_{CZ}}$. The number of CZ cells located at the periphery of the CZ at time t is denoted by $n_p(t)$. It holds $n_p(t) = 2\Pi R_{CZ}(t)/\sqrt{a_{CZ}} = 2\sqrt{n_{CZ}(t)\Pi}$. We obtain the following ordinary differential equation describing the CZ dynamics:

$$\frac{d}{dt}n_{CZ}(t) = p_{CZ}n_{CZ}(t) - n_p(t)\gamma_{CZ}(R_{CZ}(t)), \tag{1}$$

where p_{CZ} is the proliferation rate of CZ cells and γ_{CZ} depends on the CZ radius as depicted in Figure 1.

**Variable/Parameter**	**Meaning**
t	Time
$n_{CZ}(t)$	Number of CZ cells at time t
a_{CZ}	Area occupied by an average CZ cell
δ	Average diameter of a CZ cell
$A_{CZ}(t)$	Area of the CZ at time t
$R_{CZ}(t)$	Radius of the CZ at time t
p_{CZ}	Proliferation rate of CZ cells
$\gamma_{CZ}(R)$	Differentiation rate of peripheral CZ cells at distance R from the CZ center
n_p(t)	Number of CZ cells with contact to peripheral CZ boundary at time t
t_{sep}	Time elapsing between generation and separation of a primordium
$\tau(n_{CZ})$	Time elapsing between initiation of two incipient primordia
n^0_{Pr}	Size of incipient primordia at the time of initiation
$P_i(t)$	Number of cells in (incipient) primordium i at time t
$Q_i(t)$	Number of unspecified PZ cells surrounding incipient primordium i
p_{PZ}	Proliferation rate of PZ cells
γ_{PZ}	Differentiation rate of peripheral unspecified PZ cells

Table 1: List of important variables and parameters.

Dynamics in the PZ

The PZ consists of unspecified cells and of cells forming the incipient primordia. The incipient primordia are initiated at the inner boundary of the PZ. At the time of initiation each incipient primordium consists of n^0_{Pr} cells. As initiation we understand a fate transition of n^0_{Pr} cells from the unspecified PZ cell fate to the incipient primordia cell fate. We assume that the time interval τ between initiation of two primordia depends on the CZ cell number $n_{CZ}(t^*)$ at the time t^* when the latest initiation happened. We assume that each incipient primordium grows for a fixed time t_{sep} and then separates from the meristem.

We model the processes of the meristem as follows.

- The flux of cells from the CZ into the PZ at time t is given by $n_p(t)\gamma_{CZ}(R_{CZ}(t))$. Cells leaving the CZ form a ring of unspecified PZ cells surrounding the CZ, see Figure 2 (a). The number of cells entering this ring between t_0 and t_1 is given by $\int_{t_0}^{t_1} n_p(t)\gamma_{CZ}(R_{CZ}(t))dt$.

- Primordia are initiated at discrete time points inside the innermost cell layer of the PZ. During initiation n^0_{Pr} unspecified PZ cells adopt the fate of incipient primordia cells, see Figure 2 (b). We denote the incipient primordia by P_i, $i = 1 \ldots n$. Primordia are visualized such that the angle between two subsequently initiated incipient primordia is 137,5^o.

- Structures of the peripheral zone, i.e., incipient primordia and unspecified PZ cells, expand due to cell division. Each incipient primordium has the shape of a sector of a circular ring. Over time each given PZ cell is displaced in direction of the periphery due to influx of new cells from the CZ and proliferation of PZ cells, see Figure 2 (c).

- After the time interval τ an additional incipient primordium is initiated at the inner boundary of the PZ.

- We assume that unspecified PZ cells which have a higher distance from the center than cells of the outermost primordium contribute to longitudinal growth of the plant and disappear from the meristem at a constant rate γ_{PZ}, see Figure 2 (d).

- We assume that primordia separate from the meristem a fixed time t_{sep} after their initiation. Separated primordia are removed from the model system, see Figure 2 (e).

Using these assumptions we obtain a circular meristem geometry where incipient primordia are represented as sectors of circular rings, see Figure 2 (d). All dynamic processes in the SAM are summarized in Figure 3.

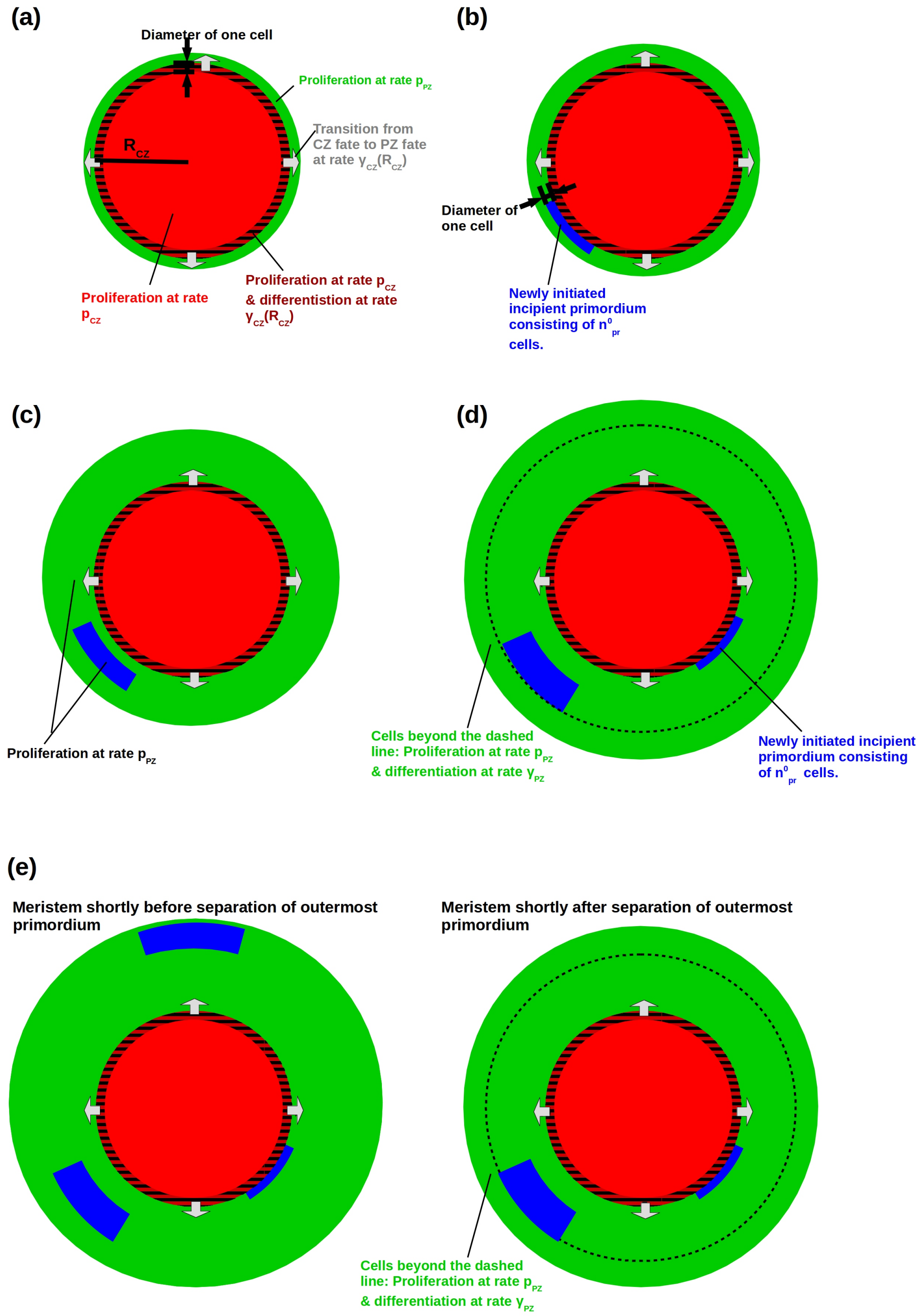

Figure 2: PZ dynamics. (a) Cell outflux from the CZ leads to a ring-shaped domain of unspecified PZ cells surrounding the CZ. (b) Primordia are initiated at the central boundary of the PZ. (c) Incipient primordia are represented as sectors of circular rings. Meristem structures are pushed towards the periphery due to cell flux from the CZ to the PZ and due to proliferation of PZ cells. (d) Unspecified PZ cells located near the periphery of the SAM contribute to longitudinal growth and disappear at a constant rate. (e) Separating primordia are removed from the system.

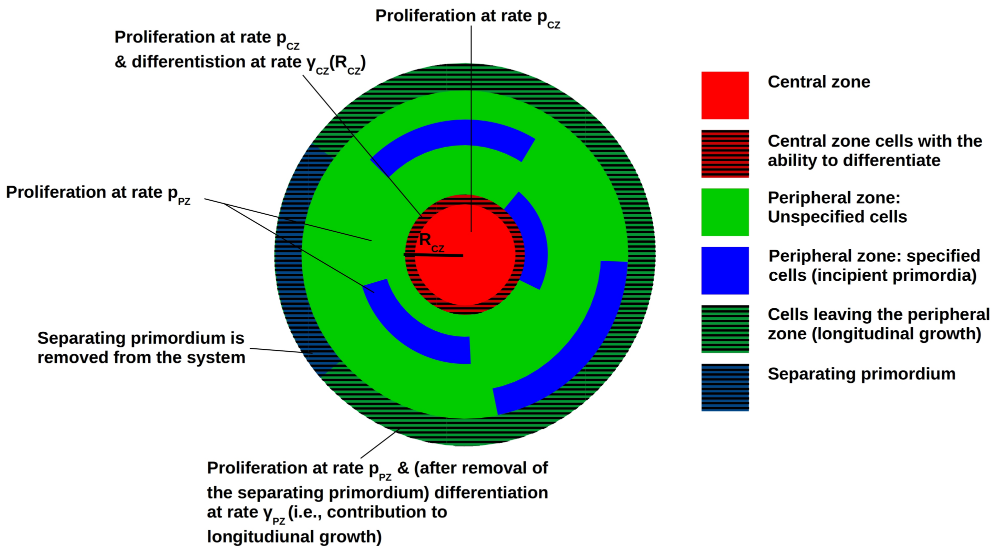

Figure 3: Summary of SAM dynamics.

Equations

The different PZ sub-populations considered in the model simulations are depicted in Figure 4. We denote incipient primordia as P_i, $i = 1, \ldots, n$, and their initiation times as t_i^*. The primordia separate from the meristem at times $t_i^* + t_{sep}$. Incipient primordia are initiated on the ring shaped domain of PZ cells which are located along the central boundary of the PZ. At the time of initiation the incipient primordium consists of $P_i(t_i^*) = n_{pr}^0$ cells. If there exist less than n_{pr}^0 unspecified PZ cells, no primordium will be initiated. The latter case is not relevant for the parameters and perturbations considered in this study.

Between the initiation of two incipient primordia, specified and unspecified PZ cells proliferate and there exists a cell flux from the CZ to the PZ. These processes lead to peripheral displacement of the incipient primordia and of the unspecified cells surrounding them. We denote by $Q_i(t)$ the number of unspecified PZ cells that have been displaced since time t_i^* from the central PZ boundary to positions inside the PZ. The progeny of those cells also belong to Q_i. These cells surround the primordium P_i as it is displaced towards the periphery.

Denote by $\eta(t_i^*)$ the number of PZ cells located at the central boundary of the PZ at time t_i^*. Directly after initiation of an incipient primordium the number of unspecified PZ cells at

the central PZ boundary equals $\eta(t_i^*) - n_{pr}^0$. It is difficult to evaluate for each time point how many of the progeny of these $\eta(t_i^*) - n_{pr}^0$ cells are located along the inner PZ boundary and how many of them have been displaced to the interior of the PZ. For simulation purposes it is sufficient to know these quantities at the time points when incipient primordia are initiated. For this reason we define a population n_u that contains all unspecified PZ cells located in a vicinity of the CZ. Evolution of n_u is given by an ODE. Only at the time points t_i^* we calculate how many of the cells from n_u are located at the boundary and how many reside in the interior of the PZ. We define n_u by $n_u(t_i^*) = \eta(t_i^*) - n_{pr}^0$ and

$$\frac{d}{dt} n_u(t) = p_{PZ} n_u(t) + n_p(t) \gamma_{CZ}(R_{CZ}(t))$$

for t between t_i^* and t_{i+1}^*. The second term describes the flux of cells from the CZ to the PZ. The population n_u consists of all unspecified PZ cells located at the inner PZ boundary at $t = t_i^*$ together with their progeny and the cell influx from the CZ.

At the time t_{i+1}^*, when the next incipient primordium P_{i+1} is initiated, we check which fraction of the population $n_u(t_{i+1}^*)$ is located at the inner PZ boundary, n_{pr}^0 of these cells will form the incipient primordium P_{i+1}. The cells from n_u which are not located at the boundary surround the incipient primordium P_i while it is pushed towards the periphery. They are denoted as Q_i. The size of the population Q_i at the time t_{i+1}^* is therefore given by $Q_i(t_{i+1}^*) = n_u(t_{i+1}^*) - \eta(t_{i+1}^*)$. This quantity is non-negative per definition.

We denote the number of cells in populations P_i and Q_i at time t by $P_i(t)$ and $Q_i(t)$ respectively. Dynamics of P_i, Q_i and S_i are given by the following ODEs:

$$\frac{d}{dt}n_{CZ}(t) = p_{CZ}n_{CZ} - n_p(t)\gamma_{CZ}(R_{CZ}(t)) \tag{2}$$

$$\frac{d}{dt}n_u(t) = p_{PZ}n_u(t) + n_p(t)\gamma_{CZ}(R_{CZ}(t)) \tag{3}$$

$$\text{for } t \in [t_i^*, t_{i+1}^*), n_u(t_i^*) = \begin{cases} \eta(t_i^*) - n_{pr}^0 & \text{if } \eta(t_i^*) - n_{pr}^0 \geq 0 \\ \eta(t_i^*) & \text{if } \eta(t_i^*) - n_{pr}^0 < 0 \end{cases}$$

$$\frac{d}{dt}P_i(t) = p_{PZ}P_i(t) \tag{4}$$

$$\text{for } t \in [t_i^*, t_i^* + t_{sep}), P_i(t_i^*) = \begin{cases} n_{pr}^0 & \text{if } \eta(t_i^*) - n_{pr}^0 \geq 0 \\ 0 & \text{if } \eta(t_i^*) - n_{pr}^0 < 0 \end{cases}$$

$$\frac{d}{dt}Q_i(t) = p_{PZ}Q_i(t) \tag{5}$$

$$\text{for } t \in [t_{i+1}^*, t_i^* + t_{sep}], Q_i(t_{i+1}^*) = \lim_{t \to t_{i+1}^* -} n_u(t) - \eta(t_{i+1}^*)$$

$$\frac{d}{dt}Q_i(t) = p_{PZ}Q_i(t) - \gamma_{PZ}Q_i(t) \tag{6}$$

$$\text{for } t > t_i^* + t_{sep}$$

$$n_p(t) = 2\sqrt{n_{CZ}(t)\Pi} \tag{7}$$

$$R_{CZ}(t) = \sqrt{n_{CZ}(t)a_{CZ}/\Pi} \tag{8}$$

The parameter p_{PZ} denotes the proliferation rate of cells located in the PZ. The rate γ_{PZ} describes the contribution of unspecified PZ cells to longitudinal growth of the plant. We set $n_{CZ}(0) > 0$.

The model is mathematically well-posed since its right hand-side is Lipschitz continuous for $n_{CZ} > 0$, what holds for all times.

A new incipient primordium is initiated by adding new equations to the system at the time of primordia initiation. At the time of primordia separation the equation describing the time dynamics of the respective primordium is removed from the system.

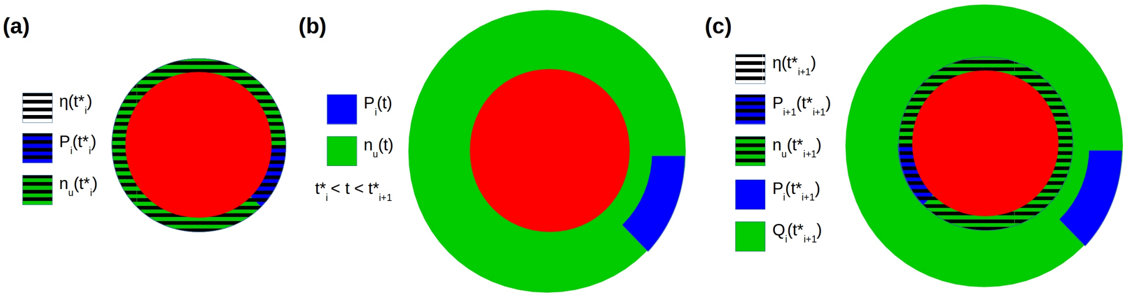

Figure 4: PZ sub-populations used for model simulation. (a) Time of initiation of the incipient primordium P_i. By $\eta(t_i^*)$ we denote the number of PZ cells located at the central boundary of the PZ. Unspecified PZ cells located at the central boundary of the PZ at the time of initiation are denoted as n_u. (b) Meristem growth is simulated until the next incipient primordium is initiated at time t_{i+1}^*. The population P_i expands due to cell division, n_u expands due to cell division and cell influx from the CZ. (c) At the time t_{i+1}^* the next incipient primordium is initiated. All cells of the population n_u from (b) which are not located at the inner PZ boundary are denoted by Q_i. They move to the periphery together with the cells from P_i. The sub-population of cells at the boundary is denoted, again, by n_u.

Calibration

We calibrate the model using the experimental data from the mock-treated plants. We assume that 1/3 of the total cells are located in L1.

CZ

We assume that the fate transition rate at the boundary of the CZ depends on the radius of the CZ. We use the function γ_{CZ} motivated above and depicted in Figure 1. Setting $r_1 = 3\delta$, $r_2 = 5\delta$ and $\hat{\gamma}_{CZ} = 2/day$ we obtain a steady state value of 43 CZ cells on L1 which is in agreement with our *in vivo* observations. Here, δ denotes the cell diameter. We notice that the value of r_2 or $\hat{\gamma}_{CZ}$ has little impact on the simulated model dynamics.

PZ

Using the cumulative number of siliques in wild type plants we infer that each 10.55 hours (0.44 days) a primordium separates from the meristem to form a silique. To obtain a constant number of unseparated primordia in steady state, a new primordium has to be initiated each 10.55 hours ($\tau = 0.44$ *days*). This choice is in agreement with data from [1,2]. According to our *in vivo* observations there exist 5 primordia in the periphery of the SAM. This implies that each primordium separates after 52.8 hours ($t_{sep} = 2.2$ *days* $= 5\tau$).

According to [6], we set $n_{pr}^0 = 4$. Proliferation rates of CZ and PZ cells have been chosen within the ranges reported in [1,2]. Nevertheless it cannot be ruled out that the rates from [1, 2] differ from the real proliferation rates since the experimental setup might have impacts on the SAM dynamics. For the depicted simulations it was assumed that CZ cells divide each 40 hours ($p_{CZ} = 0.4177/day$) and PZ cells each 18.3 hours ($p_{PZ} = 0.9064/day$). The rate γ_{PZ} is set to $5/day$. The presented results are robust with respect to the choice of this parameter.

Simulation of experimental scenarios

Expansion of WUS expressing domain

We hypothesize that in the *pCLV3:HEC1-GR* the WUS expressing domain increases in size, which leads to a reduction of CZ cell fate transition. To simulate growth of the WUS expressing domain, we assume that WUS is expressed by OC cells residing in a circular shaped domain located below the center of the SAM. We assume that the radius of this domain corresponds to the parameter r_1 in the function γ_{CZ}, see Figure 1. We assume that after induction of the *pCLV3:HEC1-GR* the radius of the WUS expressing domain increases due to increasing numbers of WUS expressing cells. We assume the number of WUS expressing cells grows at a constant rate which leads to a time dependent radius $r_1(t)$, $r_2(t) := r_1(t) + 2\delta$ and a time dependent function $\gamma_{CZ} \equiv \gamma_{CZ}(R_{CZ}, r_1(t), r_2(t))$ in the *pCLV3:HEC1-GR*. For the simulations shown the constant division rate of WUS expressing cells was set to $0.06/day$ to fit experimental observations.

Dependence of primordia initiation on CZ size

We observe that in the *pCLV3:HEC1-GR* the number of primordia is higher compared to the mock treated plants. To check the impact of time dependent primordia initiation rate on SAM dynamics, we assume that the rate of primordia initiation depends linearly on the CZ cell number. We restrict the frequency of primordia initiation to a maximum of 10 per day. We choose the slope of the linear function such that the primordia number at day 4 after induction of the *pCLV3:HEC1-GR* agrees in experimental observation and simulations. Simulation results imply that the frequency of primordia initiation has only a minor impact on the total number of CZ and PZ cells. Assuming that the initiation frequency does not depend on the CZ cell number but on the circumference of the CZ leads only to minor changes.

Re-specification of PZ cells

To rule out the re-specification of PZ cells to the CZ fate, we run simulations under the assumption that the cell flux from CZ to PZ equals zero. Simulations imply that the obtained phenotype is incompatible with experimental data. The assumption that there exists a net flux of cells from the PZ to the CZ (re-specification) leads to even stronger mismatches between data and simulations.

Hec1,2,3

To understand the steady state number of unseparated primordia in the *hec1,2,3* phenotype and the increased cumulative silique output, we simulate different scenarios. The considered scenarios include (i) higher/lower initiation frequency of primordia and unchanged separation time compared to the wildtype, (ii) unchanged initiation frequency and faster/slower separation compared to the wildtype, (iii) higher/lower initiation frequency and faster/slower separation compared to the wildtype, (iv) different initial size of incipient primordia n^0_{Pr}.

The only scenario that can explain our *in vivo* measurements is the combination of an increased initiation frequency (reduced τ) and a faster separation (reduced t_{sep}). The parameters which fit the data best are $\tau = 0.35$ *days* and $t_{sep} = 1.77$ *days*.

We infer from the HEC gain of function experiments that fate transition form the CZ to the PZ might be increased in the *hec1,2,3* phenotype. A mild increase in fate transition by 5% is in good agreement with the data. A potential reduction in PZ proliferation rate, as suggested by the HEC gain of function, seems to be mild and not necessarily required to explain the data.

The CZ transition time is estimated as follows. We assume that in the center of the CZ there exist $n_0 = 4$ stem cells which are never displaced towards the periphery. If we track a cell which is produced by one of these stem cells, we observe that it is pushed towards the periphery by offsprings of cells which have shorter distance to the center than the cell under consideration. At the time when a new cell is formed by one of the n_0 stem cells, only the n_0 stem cells have a smaller distance to the center than the newly formed cell. If we denote the steady state cell number of the CZ as $\bar{n}_{CZ}$, it takes $\log(\bar{n}_{CZ}/n_0)/p_{CZ}$ time units until the population of n_0 cells has expanded to $\bar{n}_{CZ}$ cells. This time span equals the time interval after which the considered cell is pushed from the CZ to the PZ.

.

References

[1] Reddy GV, Heisler MG, Ehrhardt DW, Meyerowitz EM. Real-time lineage analysis reveals oriented cell divisions associated with morphogenesis at the shoot apex of Arabidopsis thaliana. Development. 2004, 131(17):4225-37.

[2] Reddy GV, Meyerowitz EM. Stem-cell homeostasis and growth dynamics can be uncoupled in the Arabidopsis shoot apex. Science. 2005, 310(5748):663-7.

[3] Schoof H, Lenhard M, Haecker A, Mayer KF, Jrgens G, Laux T. The stem cell population of Arabidopsis shoot meristems in maintained by a regulatory loop between the CLAVATA and WUSCHEL genes. Cell. 2000, 100(6):635-44.

[4] Yadav RK, Perales M, Gruel J, Girke T, Jönsson H, Reddy GV. WUSCHEL protein movement mediates stem cell homeostasis in the Arabidopsis shoot apex. Genes Dev. 2011, 25(19):2025-30.

[5] Daum G, Medzihradszky A, Suzaki T, Lohmann JU. A mechanistic framework for noncell autonomous stem cell induction in Arabidopsis. Proc Natl Acad Sci U S A. 2014, 111(40):14619-24.

[6] Bossinger G, Smyth DR. Initiation patterns of flower and floral organ development in Arabidopsis thaliana. Development. 1996, 122(4):1093-102.
